# Neural basis of interindividual variability in social perception in typically developing children and adolescents using diffusion tensor imaging

**DOI:** 10.1038/s41598-020-63273-4

**Published:** 2020-04-14

**Authors:** A. Vinçon-Leite, A. Saitovitch, H. Lemaitre, E. Rechtman, L. Fillon, D. Grevent, R. Calmon, F. Brunelle, N. Boddaert, M. Zilbovicius

**Affiliations:** 1 INSERM UA10, University René Descartes, PRES Sorbonne Paris Cité and UMR 1163, Institut Imagine, Department of Pediatric Radiology, Hôpital Necker Enfants Malades, AP-HP, Paris, France; 2 Paris-Saclay University, Paris Sud University, Faculté de médecine, Paris, France

**Keywords:** Social behaviour, Brain

## Abstract

Humans show great interindividual variability in the degree they engage in social relationship. The neural basis of this variability is still poorly understood, particularly in children. In this study, we aimed to investigate the neural basis of interindividual variability in the first step of social behavior, that is social perception, in typically developing children. For that purpose, we first used eye-tracking to objectively measure eye-gaze processing during passive visualization of social movie clips in 24 children and adolescents (10.5 ± 2.9 y). Secondly, we correlated eye-tracking data with measures of fractional anisotropy, an index of white matter microstructure, obtained using diffusion tensor imaging MRI. The results showed a large interindividual variability in the number of fixations to the eyes of characters during visualization of social scenes. In addition, whole-brain analysis showed a significant positive correlation between FA and number of fixations to the eyes,mainly in the temporal part of the superior longitudinal fasciculi bilaterally, adjacent to the posterior superior temporal cortex. Our results indicate the existence of a neural signature associated with the interindividual variability in social perception in children, contributing for better understanding the neural basis of typical and atypical development of a broader social expertise.

## Introduction

Human’s everyday life is fundamentally social. Whereas social skills are very ubiquitous, the quantitative and the qualitative propensity to engage in social interactions is very heterogeneous, individual and subjective. This variability results in a wide spectrum of social behaviors, that can range from extreme shyness to extreme extroversion^1^. Further comprehension of this interindividual variability is crucial to understanding its neural substrates.

Over the last decades, brain imaging studies have largely investigated the neural basis of social processes, which allowed to describe a brain network particularly implicated in processing social information: the social brain, composed of the amygdala, the superior temporal sulcus (STS), the orbitofrontal cortex, and fusiform gyrus^2,3^. Within this network, the STS is considered a hub for social perception and social cognition, including the perception of eyes, faces and human motion, as well as understanding others’ actions and mental states^4^.

More recently, studies have also started to investigate the anatomo-functional neural correlates of interindividual variability in social functioning. Results from structural MRI studies, for instance, showed a correlation between different degrees of social behavior and gray matter volume in specific brain regions^5–8^. Functional MRI (fMRI) activation studies showed different degrees of brain activation in specific regions associated with different levels of social behavior^9–11^. Lately, resting state fMRI (rs-fMRI) studies demonstrated that intrinsic brain functional connectivity is associated with interindividual variability in cognitive performances, such as musical or reading abilities^12^. Moreover, it has been suggested that individual differences in network topography can be associated with individual phenotypes regarding cognition, personality, and emotion^13–15^.

Even though brain imaging investigations in social neurosciences have mainly focused on cortical grey matter, the major role white matter plays in communication between different brain areas indicates that investigating its association with specific functions can lead to a better understanding of human cognition and behavior^16–18^.Indeed, diffusion tensor imaging (DTI) studies have allowed to describe structural white matter correlates of various psychiatric and neurological disorders^19^, as well as an association between anatomical connectivity and individual traitsof social behavior in young healthy adults^18^. For instance, a widespread significant negative correlation was described between neuroticism traits and fractional anisotropy (FA)^20^. An association between increased reward dependency and decreased FA was also described in frontally distributed areas including pathways connecting important constituents of reward-related structural networks^21^. Finally, an association between greater levels of empathy and greater white matter integrity in fiber tracts connecting regions involved in action production and action perception, visual and affective processing, and regions within the limbic system was also described in healthy young adults^22^.

In typically developing children, few studies have investigated the structural white matter underpinnings of variations in social behaviors. Grosse Wiesmann and colleagues have described in toddlers that higher scores on a false belief task correlated with higher FA around the right temporoparietal junction, left middle temporal gyrus, right ventro-medial prefrontal cortex and right precuneus^23^. In the same perspective, FA in the left uncinate fasciculus was found to be positively correlated with face-based mental state inferences^24^. Finally, in children aged from 6 to 10 years-old, higher autistic traits were significantly associated with lower FA in a small cluster at the left superior longitudinal fasciculus^25^.

Despite an expanding literature in the field, investigations on the neural basis of interindividual variability in social processes still face methodological challenges. A main challenge concerns objectively measuring such variability, since most studies addressing this issue remain based on subjective questionnaires or self-report measurements^26,27^. Yet, objective data are crucial, for instance, to improve the sensitivity of correlation studies using brain imaging methods^28^.

Since eye-gaze cueing is one of the first and basic step to engage in social interactions, evaluating eye-gaze behavior could provide an objective indicator of interindividual variability within social engagement. Quantitative evaluation of gaze parameters is now possible thanks to eye-tracking technique. Although it has proved to be a powerful method in providing objective measures, eye-tracking has been rarely applied to the study of interindividual differences in social behavior in children with typical development. In a recent study conducted in our lab using eye-tracking, we described a large interindividual variability in gaze behavior during visualization of social scenes in young adults^29^. In addition, we observed a significant positive correlation between the number of fixations to the eyes and rest cerebral blood flow (CBF) within the right superior temporal regions. Considering that the preference for the eyes as a privileged attention target appears early in development, exploring such interindividual variability in social perception in children and adolescents could bring new light to the understanding of different developmental social profiles.

In this context, investigating the association between white matter microstructure and interindividual variability in an earliest step of social behavior, that is, social perception, in typically developing children could contribute to a better understanding of the neural circuit supporting the ongoing acquisition of an increasingly complex social behavior. Therefore, in the present study, we specifically tested two hypotheses: 1) it is possible to objectively measure interindividual variability in social perception in typically developing children and adolescents using eye-tracking and 2) this putative interindividual variability is correlated with individual differences in brain white matter microstructure, measured with DTI-MRI, particularly in tracts connecting cortical areas implicated in social cognition processes, namely the social brain network.

## Results

### Eye-tracking results

**Figure 1:**
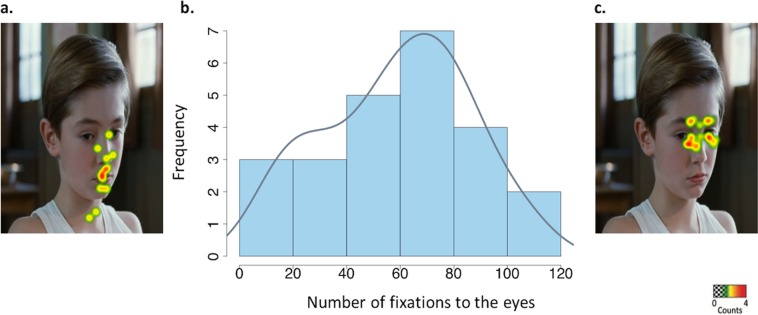
Frequency in number of fixations to the eyes in typically developing children (N = 24): a normal distribution. (**a**) and (**c**) Examples of heat map on number of fixations to the eyes: warm colors denote more fixations and cold colors denote fewer fixations to the eyes. Scenes were selected for illustrative purpose. (**b**) The histogram shows the frequency of the number of fixations to the eyes with the frequency line in blue. Images in Fig. 1 from Société Nouvelle de Distribution, used with permission.

The number of fixations to the eyes largely differed among participants (mean = 58.8; standard deviation SD = 26.6; range = 14–106) with a great variability in gaze pattern to the eyes in healthy children and adolescents when passively viewing dynamic social stimuli (kurtosis = −0.88). In addition, the number of fixations to the eyes followed a normal distribution (W = 0.96, p = 0.42, Shapiro-test) (Fig. 1). Finally, there was no correlation between number of fixations to the eyes and age of the participants (F _(1.22)_=1.9, p = 0.18, ANOVA).

## Brain imaging results

### Fractional anisotropy (FA) results

**Figure 2:**
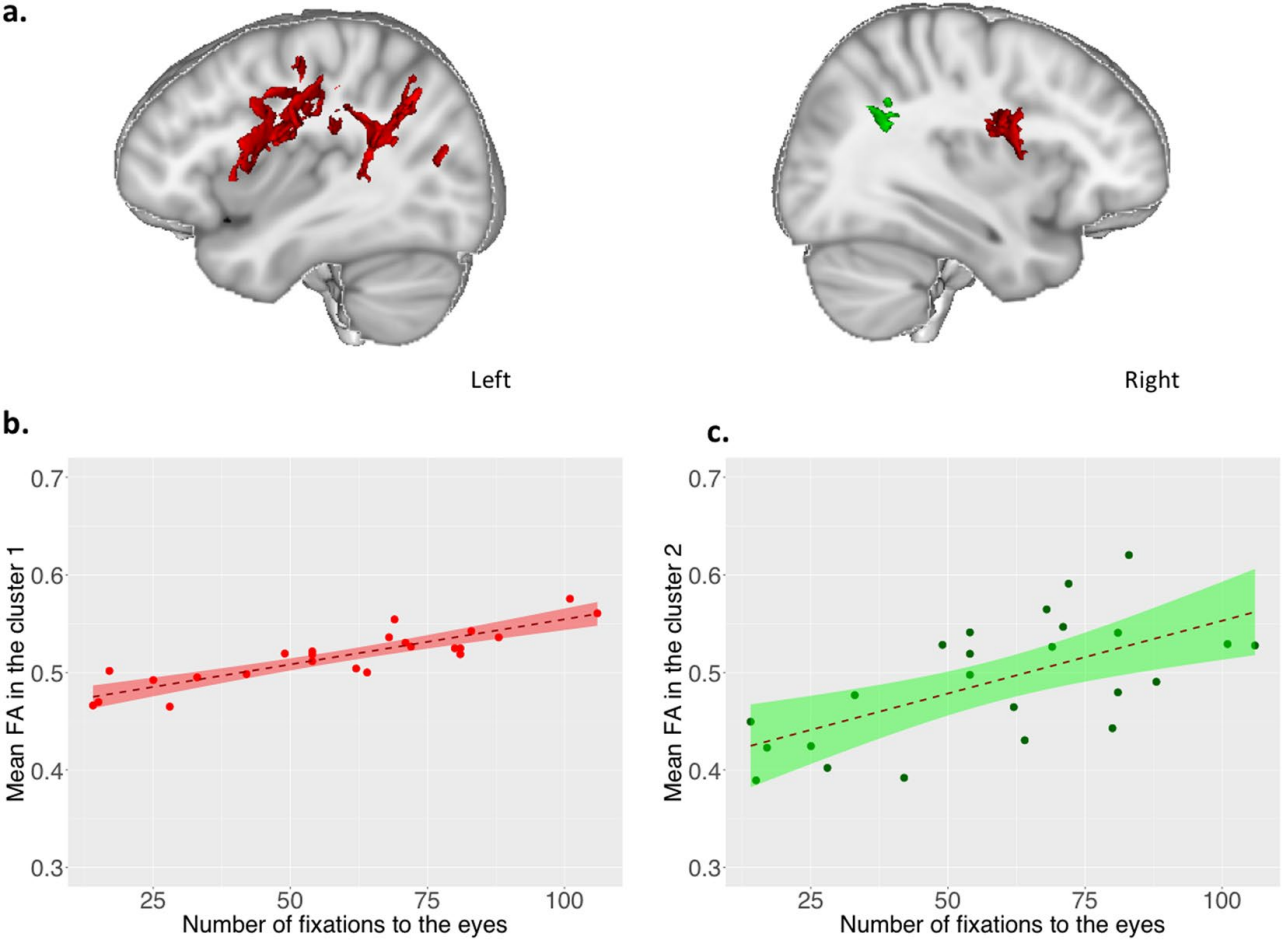
Correlation between the number of fixations to the eyes and FA values (N = 24). **(a)** Significant positive correlations between the number of fixations to the eyes and FA values within the WM skeleton (p < 0.05 Family Wise Error and Threshold Free Cluster Enhancement corrected for multiple comparisons). Results were overlaid on the MNI-152 template average brain using Mango software including the 2 significant clusters with more than 100 voxels: (cluster 1; peak voxel, x = −35, y = −50, z = 31 number of voxels=15399, p = 0.017; cluster 2, peak voxel x = 36, y = −47, z = 26, number of voxels=186, p = 0.045). Right and left sagittal views (x = 33, x = −37 respectively). **(b)** Scatterplot of positive correlation between the average FA from cluster 1 (TBSS analysis) and the number of fixations to the eyes (effect of number of fixations to the eyes b = 0.00087, t(20)=8.35, p = 5.92 e-08, regression, adding age and sex as covariates) performed in R. **(c)** Scatterplot of positive correlation between the average FA from cluster 2 (TBSS analysis) and the number of fixations to the eyes (effect of number of fixations to the eyes b = 0.0014, t(20)=3.27, p = 0.0039, regression, adding age and sex as covariates) performed in R.

Whole brain voxel-by-voxel correlation analyses between the number of fixations to the eyes and fractional anisotropy (FA) showed significant results in localized white matter tracts (p < 0.05, FWE, TFCE corrected). Within the white matter skeleton, we found a significant positive correlation between FA values and the number of fixations to the eyes in two main clusters (cluster 1: 15399 voxels, location of the maximum intensity voxel at x = −35, y = −50, z = 31, p = 0.017; cluster 2: 186 voxels, location of the maximum intensity voxel at x = 36, y = −47, z = 26, p = 0.045, Montreal Neurologic Institute coordinates). Anatomically distinct WM pathways were identifiable in these two principal cluster according to JHU White-Matter Tractography Atlas, mainly encompassing: 1) the superior longitudinal fasciculus and its temporal part localized nearby cortical temporo-parietal junction (TPJ) bilaterally; 2) the corpus callosum (forceps major); and 3) right and left anterior thalamic radiations and the left corticospinal tract (Fig. 2a, see Supplementary Table S1). Participants who looked more to the eyes of characters during passive visualization of social movies were those who had significantly higher FA values in these circumscribed WM tracts (Fig. 2b,c). In addition, we did not find any significant negative correlation between FA values and the number of fixations to the eyes. We did not find any significant positive or negative correlation between FA values and the number of fixations to the red balloon of the control scene.

### Other DTI metrics

Within the white matter skeleton, we found a significant negative correlation (p < 0.05, FWE, TFCE corrected) between radial diffusivity (RD) and the number of fixations to the eyes in 4 clusters with more than 100 voxels (cluster 1: 11245 voxels, location of the maximum intensity voxel at x = −26, y = −4, z = 23, p = 0.029; cluster 2: 293 voxels, location of the maximum intensity voxel at x = −17, y = −90, z = 9, p = 0.045, cluster 3: 198 voxels, location of the maximum intensity voxel at x = −13, y = 3, z = 52, p = 0.045; cluster 4: 107 voxels, location of the maximum intensity voxel at x = −12, y = 26, z = 49, p = 0.046, Montreal Neurologic Institute coordinates). Anatomically distinct WM pathways were identifiable in these significant clusters according to JHU White-Matter Tractography Atlas and mainly encompasses: 1) the left superior longitudinal fasciculus and its temporal part localized nearby cortical temporo-parietal junction (TPJ); 2) forceps major and forceps minor of the corpus callosum; and 3) the left corticospinal tract and left anterior thalamic radiation and 4) the left inferior longitudinal fasciculus (Supplementary Fig. S1 and Supplementary Table S1). Participants who looked more to the eyes of characters during passive visualization of social movies were those who had significantly lower RD values in these circumscribed WM tracts. In addition, we did not find any significant positive correlation between RD values and the number of fixations to the eyes. No correlation was found with axial diffusivity (AD) nor with mean diffusivity (MD).

### Commonality analysis

Since variations in white matter connections can be associated with age and gender^30,31^, to further investigate the impact of these factors in our results, we performed a commonality analysis with number of fixations to the eyes, age and gender as predictors to disentangle the contributions of each of them on the mean FA and mean RD values.

Results showed that number of fixations to the eyes alone explains 75.9% of the variance of mean FA values, whereas age alone and gender alone explain 5.5 and 1.8% of the variance, respectively (see Table 1). In addition, number of fixations to the eyes alone explains 61.9% of the variance of mean RD values, whereas age alone and gender alone explain 12.8 and 0.02% of the variance, respectively (see Table 2).

**Table 1:** Commonality analysis on FA results - Table presenting commonality coefficients and percentage of variance for each effect.

FA	Coefficient	% Total
Unique to FC eyes	0.6229	75.90
Unique to age	0.0451	5.49
Unique to gender	0.0149	1.81
Common to FC eyes and age	0.1527	18.61
Common to FC eyes and gender	−0.0119	−1.45
Common to age and gender	−0.0047	−0.57
Common to FC eyes, age and gender	0.0017	0.21
Total	0.8207	100.00

FC eyes: Fixation count to the eyes.

**Table 2:** Commonality analysis on RD results- Table presenting commonality coefficients and percentage of variance for each effect.

RD	Coefficient	% Total
Unique to FC eyes	0.5212	61.90
Unique to age	0.1079	12.82
Unique to gender	0.0002	0.02
Common to FC eyes and age	0.1907	22.65
Common to FC eyes and gender	0.0056	0.67
Common to age and gender	0.0021	0.25
Common to FC eyes, age and gender	0.0143	1.69
Total	0.8420	100.00

FC eyes: Fixation count to the eyes.

## Discussion

To the best of our knowledge, this is the first study that investigated interindividual variability in gaze behavior in typically developing children and adolescents,and its association with brain white matter microstructure. Our results indicate a large interindividual variability in social perception during visualization of social scenes in typically developing children and adolescents. Indeed, during passive visualization of video clips displaying naturalistic social scenes, some individuals look more to the eyes of characters while others look less. Moreover, such variability varies along a continuum, suggesting a “spectrum of normality” within social perception. Finally, as hypothesized, objectively measuring interindividual differences in this basic step of social behavior allows to reveal correlations with brain white matter microstructure particularly within temporal-parietal regions.

Social expertise is largely based on social perception and the importance of eye-gaze perception in broader social interactions has been largely established^32–35^. Indeed, eye contact helps infer the intentions and feelings of the conspecifics, which is crucial for survival and social integration^36^. Moreover, the preference for the eyes as a privileged attention target is evident extremely early in the normal development, suggesting that this preference is a core mechanism for the subsequent development of a larger expertise of human social cognition. Interestingly, it has been shown that the ability to process eye gaze information is negatively correlated with self-reported loneliness^7^. In addition, eye-tracking studies have shown that visual patterns while watching emotionally loaded images may depend on personality traits^37,38^. Here, rather than describing variability based on subjective measures of complex social behavior, our results point towards interindividual differences in a basic process that is already present from an early stage of development and thus is a key component of social behavior.

Our findings provide new insights to the understanding of between subjects’ developmental differences in broader social behavior. In addition, they suggest the existence of an individual signature regarding social behavior during typical development, which could be associated with different developmental trajectories towards social expertise. One can imagine that children who look less to the eyes will have a different developmental trajectory regarding social expertise than children who look more to the eyes, resulting in different social profiles. Longitudinal studies remain needed to confirm this hypothesis.

Previous studies focusing on white matter correlates of social cognition processes in children have mainly used questionnaires and subjective tasks^23–25^. In this context, eye-tracking investigations could provide more objective and quantitative measures. Even though it has proved to be a powerful tool to investigate social perception abnormalities in various neurodevelopmental disorders^39–42^, outside the scoop of pathology it had so far not been used to explore gaze behavior in social contexts in typically developing children.

In this study, we describe white matter microstructure correlates of interindividual variability in eye-gaze perception within fronto-temporal pathways comprising the superior longitudinal fasciculi (SLF), mainly its temporal part. Children who looked more to the eyes of characters were those presenting higher FA values in these tracts, which lie in close vicinity of the superior temporal gyrus, the angular gyrus and the temporal-parietal junction. Coherently with FA results, we found a significant negative correlation between individual number of fixations to the eyes and RD values roughly in the same areas. The superior longitudinal fasciculus connects superior posterior face-selective regions, such as the STS, with anterior inferior face-selective regions (inferior frontal gyrus and orbitofrontal cortex)^43^. Functionally, the SLF has been associated with gaze processing^44^.

An association between individual patterns of social perception and white matter microstructure bounded to the superior temporal regions becomes also relevant in light of current consensus on the major cortical role of these regions in social cognition^2,4,45^. A large number of functional MRI (fMRI) studies has shown that the superior temporal regions, mainly the posterior superior temporal sulcus (pSTS), is highly implicated in processing social information, mostly conveyed by the eyes^46,47^. Additionally, correlations between WM and number of fixations to the eyes were also observed in the corpus callosum and in the left corticospinal tract, which could be related to oculo-motor aspects of gaze behavior.

It’s important to highlight some limits of the present study. Firstly, it has been previously shown that white matter connections change with age and gender and those factors could partially account for the results. The commonality analysis we performed do not indicate an age effect in our dataset, since number of fixations to the eyes alone explained over 75% of the variance of the mean FA. Replication studies in a larger cohort are nevertheless needed to confirm these findings. In addition, even though FA variations point to variations in axonal density, membrane integrity, and/or myelination^31^ as candidate mechanisms underlying the interindividual difference in social perception, the neurobiological underpinnings of diffusion properties in the brain are complex and interpretation should remain cautious^48^.

Taking a step further into the understanding of brain-behavior association in typical development, our findings indicate not only a great variability in social perception in typically developing children but also that this variability is linked to individual patterns of white matter microstructure. If confirmed, these results could provide a structural mechanism for variability in social perception in children and open up a framework for better understanding the neural basis of typical and atypical development of a broader social expertise.

## Materials and Methods

### Participants

Twenty-four typically developing children and adolescents (15 males, 9 females; mean age=10.5 years, standard deviation SD = 2.9 years; range: 6.0 to 17.4 years) participated in this study. All participants were free of psychiatric, neurological and general health problems, as well as any learning disabilities. All of them have a normal scholarship. All participants presented no contraindication for the MRI scan and had normal vision. All participants were volunteers, recruited by advertisement. Written informed consent to participate to this study was obtained from each participant parents or legal guardians and adhere to the principles of the Helsinki Declaration. The study was approved by the Ethical Committee of Necker Hospital. All experiments and methods were performed in accordance with relevant guidelines and regulations.

#### Eye-tracking protocol

The study was performed using the Tobii T120 eye-tracker equipment, based on infra-red technology, consisting of a 17-inch TFT monitor with a resolution of 1280 × 1024 pixels, from which the stimuli were presented in full screen, and the gaze behavior was simultaneously recorded. The eye-tracking system was completely non-invasive with little indication that the eye-movements were being tracked. No artificial constraints of the head or body movements were necessary. The system tracked both eyes with an accuracy of 0.5 degrees and a sampling rate of 60 Hz. The Tobii equipment was connected to an HP pavillon dv6 laptop computer (Windows 7 Professional).

The participants were individually tested, seated facing the eye-tracker monitor at approximatively 60 cm; the experimenter sat next to the participant to control the computer without interfering with the viewing behavior. A calibration test consisting of five registration points was performed before each set of stimuli. The calibration test was repeated if the examiner considered one of the five points not valid according to the eye-tracker criteria. The participants were instructed that they would see a sequence of movie fragments and that all they had to do was to watch them. The stimuli creation, the calibration procedures and the data acquisition and visualization were performed using Tobii Studio software.

#### Stimuli

To investigate the individual variability in eye-gaze behavior across a group of healthy children and adolescent aged from 6 to 17 years, we used an eye-tracking paradigm developed in our lab^29,49^. The eye-tracking task was a passive visualization of naturalistic social movies; no specific task performance was required. To provide an ecological and naturalistic setting, we used short movie fragments (10 seconds) extracted from commercial films (25 fps). Five fragments displayed social scenes with 2 characters engaged in peer to peer social interactions (Le Petit Nicolas) and 2 fragments displayed a simple nonsocial scene with a red balloon flying against a blue sky (Le ballon rouge), to control for changes linked to the perception of nonbiological movement. Sounds in the movies were dialogs in the social scenes and soft music in the nonsocial scenes. Factors as scene background, characters’ position, balloon size, or speed were not controlled for.

MRI acquisition: Separately from the eye-tracking session, all participants underwent an MRI scan in which diffusion tensor imaging (DTI) was used to measure fractional anisotropy (FA), as an index of brain white matter (WM) microarchitecture as well as mean diffusivity (MD), radial diffusivity (RD) and axial diffusivity (AD). All brain images were acquired with a GE-Signa 1.5 Tesla MR scanner located at Necker Hospital, in Paris, using a 12-channel head coil. Clinical sequences were acquired (3DT1, coronal T2 and coronal FLAIR) allowing us to ensure participants presented no radiological brain abnormalities. Diffusion-weighted images were acquired using an echo-planar imaging sequence (axial slices, echo time (TE)≈ 96 ms, repetition time (TR)= 15 000 milliseconds, 40 diffusion encoding directions with b-value=1000 s/mm^2^ and one b-value=0 s/mm^2^, voxel size: 2.4 × 2 × 2 mm), adapted to tensor measurements.

#### Diffusion imaging preprocessing

First, all raw diffusion images were visually checked for major artefacts. Then, diffusion data were preprocessed using the DESIGNER pipeline^50^ with tools available in MRtrix3 package^51^ in order to denoise, dering and bias correct the raw images. Indeed to reduce the noise effect on the diffusion parameter estimation, the MRtrix3 *dwidenoise* tool (Copyright ^©^2016 New York University, University of Antwerp, https://github.com/MRtrix3/mrtrix3) was applied as the first step of the preprocessing^52,53^. Then, the Gibbs ringing correction framework of Kellner *et al.*^54^ was applied to remove Gibbs ringing artifacts with the *mrdegibbs* tool. Finally, we applied B1 field inhomogeneity correction^55^ using the *dwibiascorrect* tool. After denoise, degibbs and bias correct, images were corrected for head motion and eddy‐currents using FMRIB Diffusion Toolbox (FDT) in FMRIB Software Library (FSL) (www.fmrib.ox.ac.uk/fsl)^55^. It consisted of an affine registration to the first b = 0 image for head motion and eddy currents corrections. Gradient tables were reoriented accordingly to the affine transformation. Then, we performed a brain extraction using the Brain Extraction Tool (BET), and a voxel-wise diffusion tensor fitting to obtain images of Fractional Anisotropy (FA), mean diffusivity (MD), radial diffusivity (RD) and axial diffusivity (AD). Furthermore, for quality improvement, we performed Robust Estimation of Tensors by Outlier Rejection (RESTORE) on a voxel-wise basis to identify and exclude outliers from the multiple diffusion directions collected, and to calculate the diffusion tensor from the remaining data to provide more robust estimates of diffusion parameters than other fitting procedures^56^. An automatic quality control was performed during the preprocessing to detect large head motion and signal dropout. One participant (1/25) was discarded because of head motions and blink eye artefacts.

#### Tract-based spatial statistics

Voxel-wise statistical analysis of the FA data was carried out using Tract-based spatial statistics (TBSS)^57^, part of FSL. Every FA image was align to every other one, identifying the “most representative” one, and using this as the target image. This target image was then affine-aligned into MNI152 standard space, and every image was transformed into 1 × 1 × 1 mm MNI152 space by combining the nonlinear transform to the target FA image with the affine transform from that target to MNI152 space. Next, the mean FA images were created and thinned to create a mean FA skeleton, which represents the center of all tracts common to the group. This skeleton was then threshold to FA > 0.2 to keep only the main tracts. Each children’s and adolescent’s aligned FA, MD, RD, AD maximum values were then projected onto the skeleton and the resulting data fed into voxel-wise cross-individual statistics. A visual quality control was performed on the FA images for proper spatial normalization and brain masking. The final sample for the TBSS analysis was constituted of 24 participants.

### Statistical analysis

#### Eye-tracking

Gaze patterns were analyzed with dynamic area of interest (AOI) allowing “frame by frame” measurements throughout the film. For each movie fragment, the dynamic AOI selected for analysis was the eyes region of the characters in the social movie fragments and the balloon in the non- social movie fragments. Eye-tracking software interpolates the shape and position of the AOI, so that it moves smoothly from one frame to the next. More importantly, AOIs sizes and shapes remained stable across measurements. The number of fixations in the AOI “eyes” and “balloon” was recorded using the Tobii Studio software. A fixation event was defined as such by the Tobii fixation filter based on 0.42 pixels/ms threshold. Number of fixations was selected since it is an absolute variable that informs on exploratory behavior toward a defined region: higher number of fixations indicates that people further explore the region. A threshold of 80% of valid data was stablished and all participants included in this study matched such recording quality criteria, based on the amount of valid and missing data, as indicated by Tobii studio software.

The number of fixations to the eyes was extracted and then exported to R cran software (http://www.R-project.org/). Normality of the number of fixations to the eyes was tested using the Shapiro-test. The Pearson measure of kurtosis was derived from the distribution of the number of fixations to the eyes.

#### DTI-MRI

Voxel-wise correlations between number of fixations to the eyes and FA, MD, RD, AD were tested within the framework of the general linear model (GLM) using a randomization-based method (10.000 permutations). Independent analyses were conducted including each DTI metrics as the dependent variables, and the number of fixations to the eyes as the independent variable. Statistical thresholds were set at p < 0.05 corrected for family-wise error (FWE) and we used the Threshold-Free Cluster Enhancement (TFCE) option^58^. Only clusters with an extent >100 continuous voxels were considered to eliminate isolated small clusters from further consideration. The Johns Hopkins University (JHU) tractography atlas^59^ were used to locate the tracts that displayed significant correlations.

The mean FA from the clusters showing significant correlations with eye-fixation were extracted and then exported to R cran software. Same linear regressions between the number of fixations in the eyes and extracted mean FA values in the significant clusters were also performed within the framework of the general linear model adding age and gender as covariate.

#### Commonality analysis

To further investigate the impact of age and gender on mean FA and mean RD values, we performed a commonality analysis in Rcran software^23,60,61^. A commonality analysis allows to decompose the contribution of different predictors into subcomponents explained by the unique variance of the individual predictors, or the shared variance of combinations of the predictors. Therefore, with the commonality analysis, we can disentangle whether the FA/RD variations were explained by unique contribution of individual differences in number of fixations to the eyes, age and gender or by a combination of the different factors.

## Supplementary information

Supplementary information.

## Data Availability

The data that support the findings of this study are available on request from the corresponding author V-L A. The data are not publicly available due to data protection policy in the ethics agreement.
