# Supplementary material for: Neural basis of interindividual variability in social perception in typically developing children and adolescents using diffusion tensor imaging
Source: Sci Rep. 2020 Apr 14;10:6379. doi: 10.1038/s41598-020-63273-4 (PMC7156418; doi:10.1038/s41598-020-63273-4)

Supplementary Information

Article: Neural basis of interindividual variability in social perception in typically developing children and adolescents using diffusion tensor imaging

AUTHORS NAMES

A. Vinçon-Leite[1], A. Saitovitch[1], H. Lemaitre[1,2], E. Rechtman[1], L. Fillon[1], D. Grevent[1], R. Calmon[1], F. Brunelle[1], N. Boddaert[1] and M. Zilbovicius[1]

Supplementary Table S1: Correlation between DTI metrics (FA and RD) and number of fixations to the eyes (N= 24 healthy children)

[a] Mean probabilities of the considered tract within the mask of results (outputs from 'autoaq' of FSL) in order of importance

Variable	Voxel size	White-matter tracts JHU white matter tractography atlas	Mean probabilities[a]
**FA**			
Cluster 1	15399	Superior longitudinal fasciculus L	5,08
		Anterior thalamic radiation R	2,18
		Anterior thalamic radiation L	2,07
		Superior longitudinal fasciculus (temporal part) L	2,05
		Corticospinal tract L	1,74
		Forceps major	1,26
		Forceps minor	0,91
		Cingulum (cingulate gyrus) L	0,79
		Inferior fronto-occipital fasciculus L	0,58
		Inferior longitudinal fasciculus L	0,51
		Superior longitudinal fasciculus R	0,485
		Inferior fronto-occipital fasciculus R	0,24
		Corticospinal tract R	0,17
		Superior longitudinal fasciculus (temporal part) R	0,11
		Uncinate fasciculus L	0,065
		Cingulum (cingulate gyrus) R	0,02
		Cingulum (hippocampus) L	0,02
		Uncinate fasciculus R	0,01
Cluster 2	186	Superior longitudinal fasciculus R	16,12
		Superior longitudinal fasciculus (temporal part) R	2,52
		Inferior longitudinal fasciculus R	0,54
		Inferior fronto-occipital fasciculus R	0,11
		Anterior thalamic radiation R	0,03
		Cingulum (hippocampus) R	0,02
**RD**			
Cluster 1	11245	Superior longitudinal fasciculus L	6,77
		Superior longitudinal fasciculus (temporal part) L	2,73
		Anterior thalamic radiation L	2,25
		Corticospinal tract L	2,03
		Forceps minor	1,72
		Inferior longitudinal fasciculus L	1,03
		Forceps major	0,72
		Inferior fronto-occipital fasciculus L	0,60
		Cingulum (cingulate gyrus) L	0,58
		Uncinate fasciculus L	0,55
		Anterior thalamic radiation R	0,31
		Cingulum (cingulate gyrus) R	0,02
		Corticospinal tract R	0,02
		Superior longitudinal fasciculus (temporal part) R	0,01
		Cingulum (hippocampus) L	0,01
		Superior longitudinal fasciculus R	0,01
Cluster 2	293	Forceps major	20,57
		Inferior fronto-occipital fasciculus L	5,97
		Inferior longitudinal fasciculus L	5,65
		Anterior thalamic radiation L	0,32
		Cingulum (hippocampus) L	0,19
		Superior longitudinal fasciculus L	0,05
		Superior longitudinal fasciculus (temporal part) L	0,05
Cluster 3	198	Superior longitudinal fasciculus L	0,09
		Superior longitudinal fasciculus (temporal part) L	0,045
Cluster 4	107	Uncinate fasciculus L	0,25
		Cingulum (cingulate gyrus) L	0,22
		Superior longitudinal fasciculus L	0,20
		Superior longitudinal fasciculus (temporal part) L	0,11

Supplementary Figure S1: Correlation between the number of fixations to the eyes and RD values in typically developing children (N= 24).

a, c, e, g: Significant negative correlations between the number of fixations to the eyes and RD within the WM skeleton ($p < 0.05$ Family Wise Error and Threshold Free Cluster Enhancement corrected for multiple comparisons). Results were overlaid on the MNI-152 template average brain using Mango software including 4 significant clusters with more than 100 voxels (cluster 1: 11245 voxels, location of the maximum intensity voxel at x = -26, y =-4 , z =23, p = 0.029 ; cluster 2: 293 voxels, location of the maximum intensity voxel at x = -17, y =-90 , z = 9, p = 0.045 , cluster 3 : 198 voxels, location of the maximum intensity voxel at x = -13, y =3, z =52, p = 0.045 ; cluster 4: 107 voxels, location of the maximum intensity voxel at x = -12, y =26 , z = 49, p = 0.046 , Montreal Neurologic Institute coordinates). a, c, e, g, sagittal views (x= 11 and x = -38, x = -10, x=-10, x = -8 respectively)

b, d, f, h: Scatterplot of negative correlation between the average RD from the four cluster with more than 100 voxels (TBSS analysis) and the number of fixations to the eyes (effect of number of fixations to the eyes, cluster 1: b = -9.86e-07, t(20)= -7.99, p = 1.2e-07; cluster 2: b = -9.5e-07, t(20)= -5.5, p = 2.21e-05; cluster 3: b = -1.29e-06, t(20)= -4.83, p = 0.0001; cluster 4: b -1.52e-06=, t(20)= -5.55, p = 1.95e-05; regression adding age and sex as covariates).

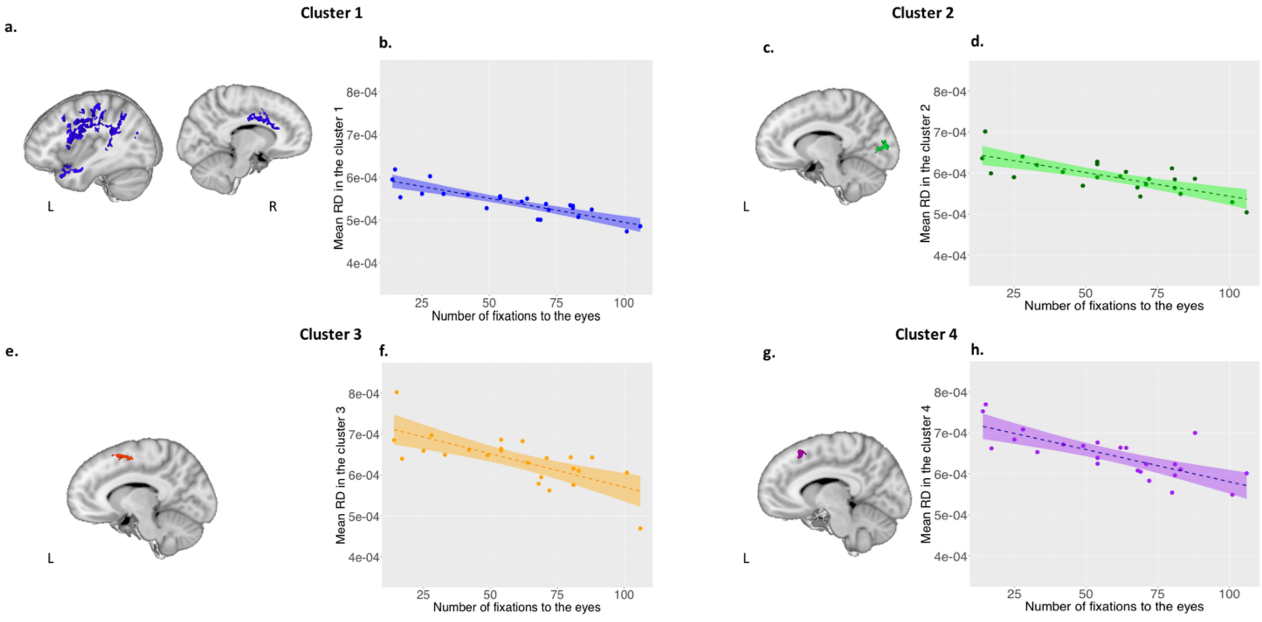

Supplement: Supplementary file 1 — Supplementary information. [file 41598_2020_63273_MOESM1_ESM.pdf]
